# A27 COMPARATIVE EVALUATION OF THE ABC SCORE TO OTHER RISK STRATIFICATION SCALES IN MANAGING HIGH-RISK PATIENTS PRESENTING WITH ACUTE UPPER GASTROINTESTINAL BLEEDING

**DOI:** 10.1093/jcag/gwab049.026

**Published:** 2022-02-21

**Authors:** A N Barkun, O Kherad, S Restellini, M Almadi, M Martel

**Affiliations:** 1 Gastroenterology, McGill University, The Montreal General Hospital, GI Division, Montreal, QC, Canada; 2 Hôpital de la Tour and University of Geneva, Geneva, Switzerland; 3 Geneva University Hospitals and University of Geneva, Geneva, Switzerland; 4 King Khalid University Hospital, King Saud University, Riyadh, Saudi Arabia

## Abstract

**Background:**

The ABC risk score identifies patients at high-risk of mortality in acute lower and upper gastro-intestinal bleeding (UGIB).

**Aims:**

We aimed to externally validate the ABC score, while comparing it to other prognostication scales when assessing UGIB patients at high-risk of negative outcomes prior to endoscopy.

**Methods:**

UGIB patients from a national Canadian registry (REASON) were studied, with mortality prediction as primary outcome. Secondary endpoints included prognostication of rebleeding, intensive care unit (ICU) admission, ICU and hospitalization lengths of stay (LOS), and a previously proposed composite outcome measure. Univariable and areas under the Receiver Operating Characteristic Curve (AUROC) analyses compared discriminatory abilities of the ABC score to the AIMS65, Glasgow Blatchford (GBS) and clinical Rockall Scores.

**Results:**

The REASON registry included 2020 patients (89.4% nonvariceal; mean age [± SD] 66.3±16.4 years; 38.4% female). Overall mortality, rebleeding, ICU admission, transfusion and composite score rates were 9.9%, 11.4%, 21.1%, 69.0%, and 67.3% respectively. ICU and hospitalization LOS were 5.4 ± 9.3 days and 9.1 ± 11.5 days, respectively. The ABC score displayed superior 30-day mortality prediction (0.78 (0.73; 0.83)) compared to GBS (0.69 (0.63; 0.75) or clinical Rockall (0.64 (0.58; 0.70) but not AIMS65 (0.73 (0.67; 0.79)). Although most scales significantly prognosticated secondary outcomes in univariable analysis except for ICU LOS, discriminatory abilities on AUROC analyses were poor.

**Conclusions:**

ABC and AIMS65 display similar good prediction of mortality. Clinical usefulness in prognosticating secondary outcomes was modest for all scales, limiting their adoptions when informing early management of high-risk UGIB patients.

**Funding Agencies:**

International Scientific Partnership Program ISPP at King Saud University for funding this research work through ISPP-21–156

